# CT-Guided Lung Biopsy Using Dual-Energy Iodine Mapping to Target Lung Masses with Necrotic Tissue—A Proof-of-Concept Study

**DOI:** 10.3390/jcm15041415

**Published:** 2026-02-11

**Authors:** Eviatar Naamany, Eli Atar, Mordechai Reuven Kramer, Reut Anconina, Lutof Zreik, Lev Freidkin, Barak Pertzov, Osnat Shtraichman, Shai Moshe Amor

**Affiliations:** 1 Pulmonary Division, Rabin Medical Center, Petah Tikva 4941492, Israel; 2 Gray Faculty of Medical & Health Sciences, Tel Aviv University, Tel Aviv-Yafo 6997801, Israel; 3 Radiology Department, Rabin Medical Center, Petah Tikva 4941492, Israel; 4 Department of Medical Imaging, Rambam Health Care Campus, Haifa 3109601, Israel

**Keywords:** dual-energy CT, iodine mapping, precision oncology, CT-guided biopsy, functional imaging

## Abstract

**Background:** Computed tomography (CT)-guided lung biopsy plays a pivotal role in diagnosing thoracic lesions. However, its diagnostic yield may be compromised in large, necrotic, or heterogeneous tumours due to inadvertent sampling of non-viable tissue. Dual-energy CT (DECT) iodine mapping provides functional imaging by identifying iodine-avid, perfused areas, thereby offering the potential to improve biopsy targeting. **Methods:** This single-centre retrospective study evaluated the clinical feasibility and diagnostic performance of DECT-guided biopsy. Adult patients with suspected necrotic lung or mediastinal lesions who underwent DECT iodine mapping prior to CT-guided biopsy between April 2021 and December 2022 were evaluated. DECT iodine maps were generated using dual-source CT and used to identify viable tumour regions for targeted biopsy. The primary outcome was diagnostic yield, defined as obtaining a definitive histopathological diagnosis. Secondary outcomes included safety and adequacy of samples for molecular testing. **Results:** Twenty patients were included. A definitive diagnosis was obtained in 18/20 biopsies (90%). Diagnostic yield was 9/11 (81.8%) for pulmonary lesions and 9/9 (100%) for mediastinal/pleural lesions. Diagnoses included non-small-cell lung cancer (n = 8), Hodgkin lymphoma (n = 4), thymoma (n = 3), and other malignancies (n = 3). Biopsy material was sufficient for additional molecular testing in 13/20 cases (65%). Complications were minor (one pneumothorax not requiring drainage and two self-limited bleeding events). **Conclusions:** DECT iodine map-guided targeting was feasible in this retrospective cohort and was associated with high diagnostic yield, low complication rates, and frequent acquisition of tissue suitable for molecular analyses. Prospective controlled studies are needed to quantify benefit over conventional CT guidance.

## 1. Introduction

Lung cancer remains the leading cause of cancer-related death worldwide [1]. Accurate tissue diagnosis is critical for distinguishing between benign and malignant pulmonary lesions and guiding effective treatment. Although imaging modalities such as CT and positron emission tomography (PET) provide meaningful insights into lesion morphology and metabolism [2,3], histopathologic confirmation remains essential.

Traditionally, diagnosis focused on differentiating small-cell lung cancer (SCLC) from non-small-cell lung cancer (NSCLC). However, the growing importance of molecular subtyping in NSCLC heightened the need for high-quality biopsy tissue samples [4].

CT-guided core needle biopsy (CTGB) is a widely accepted, accurate method for sampling pulmonary and mediastinal lesions, demonstrating high diagnostic performance with a reported accuracy of 90–100% [5,6,7]. Nonetheless, the false-negative rate could increase significantly in large, heterogeneous lesions, particularly those with cystic and necrotic areas, due to the limited sampling obtained by CTGB. To address this, techniques like intra-procedural CT or PET/CT fusion imaging have been explored. However, PET/CT may not always be available or up to date, limiting its utility for real-time biopsy targeting [8].

Dual-energy CT (DECT) offers advanced tissue characterisation by generating iodine maps based on material-specific attenuation differences [9]. These maps enhance lesion visualisation, particularly for viable tumour tissue, by highlighting areas of contrast uptake. Over recent years, numerous applications for DECT have emerged, including the enhancement of liver tumour detection [10] and expediting abdominal or cardiac post-processing; for example, dual-energy–based post-processing has been shown to reduce blooming and beam-hardening artefacts from calcified plaques and to maintain accurate plaque quantification even under simulated cardiac motion, supporting the technique’s robustness in dynamic conditions [11].

One of the most promising applications of DECT is the generation of virtual monoenergetic images, which can significantly improve image contrast and reduce artefacts. This technique enhances the visualisation of iodinated contrast uptake, thereby improving the delineation of vascular structures and potentially viable tumour tissue. Lenga et al. highlighted the utility of monoenergetic imaging in cardiothoracic applications, demonstrating that this approach not only enhances image quality but also reduces the required contrast dose [12].

While several studies have explored the diagnostic advantages of DECT in various oncologic and interventional contexts, its use in guiding percutaneous lung biopsy remains largely unexplored. DECT’s ability to differentiate tissue composition by analysing iodine uptake has shown promising results in tumour detection, treatment planning, and biopsy targeting in other organs. For example, DECT electron density mapping has successfully identified high-cellularity regions in bone metastases, improving diagnostic precision during CT-guided bone biopsy [13]. Similarly, dual-energy protocols have been evaluated in experimental liver biopsy models and have been shown to improve artefact reduction and the visualisation of lesion margins, although these findings have not been specifically demonstrated in necrotic tumours or clinical settings [14].

Recent reviews highlight the growing role of DECT in oncology for tumour detection, staging, and response assessment [15]. Iodine quantification maps derived from DECT can help localise viable tumour regions within heterogeneous lesions, a feature that is especially valuable for avoiding necrotic areas during biopsy. Despite this potential, no published study has demonstrated the routine use of DECT iodine maps in clinical interventional workflows for lung mass biopsy.

This study aims to provide the first clinical proof-of-concept for using DECT iodine maps for CT-guided biopsies of lung and mediastinal masses with suspected necrosis. By selectively targeting regions with high iodine uptake—indicative of viable tissue—this approach aims to improve diagnostic accuracy, reduce false-negative results, and enhance tissue adequacy for molecular testing. This study represents a novel step toward incorporating DECT into routine interventional radiology practice.

## 2. Methods

### 2.1. Study Design and Setting

This study was a retrospective single-centre study conducted at the Pulmonary Division of the Rabin Medical Center, a tertiary care academic hospital. The study aimed to evaluate the clinical utility of DECT iodine mapping in guiding CT-guided lung and mediastinal biopsies for lesions with suspected necrotic components.

This retrospective study analysed imaging, pathology, and clinical data collected during routine care for patients undergoing CT-guided biopsy of thoracic lesions. Dual-energy CT acquisition and iodine mapping were performed for enhanced imaging analysis, and CT-guided core biopsy and procedural workflow were performed as a standard of care; patient management was not altered.

### 2.2. Study Population

All consecutive patients who underwent CTGB at Rabin Medical Center between April 2021 and December 2022 were retrospectively screened for eligibility.

Patients were considered for inclusion if pre-procedural imaging demonstrated a pulmonary or mediastinal mass larger than 4 cm with suspected necrosis or internal heterogeneity on contrast-enhanced CT or Fluorodeoxyglucose positron emission tomography (FDG-PET)/CT. These criteria were chosen to enrich the cohort for lesions in which conventional CT guidance is more likely to yield non-diagnostic sampling.

### 2.3. Inclusion and Exclusion Criteria

Eligible patients were those 18 years of age or older who underwent CT-guided biopsy of a lung or anterior mediastinal mass and had dual-energy CT iodine maps available before the procedure.

Patients were excluded if:

1. Pre-procedural imaging did not show necrosis or a heterogeneous internal structure, or
2. Dual-energy CT with iodine map reconstruction was not performed prior to biopsy.

Following the application of these criteria, 20 patients constituted the final study cohort.

### 2.4. DECT Acquisition Protocol and Iodine Map Reconstruction

All patients underwent dual-source DECT using a SOMATOM^®^ Dual Source Force™ scanner (Siemens Healthineers, Forchheim, Germany). Scanning was performed at a fixed tube potential of 90 kVp, with a rotation time of 250 ms and a pitch of 0.55. Dual-source tube currents ranged from 24/46 to 390/60 mAs. Radiation exposure parameters included the average dose-length product (DLP), which was 194.6 mGy·cm (range 68.2–665.1), and the mean CTDIvol, which was 5.15 mGy (range 1.65–17.2).

Intravenous contrast (80–85 mL iohexol) was administered, followed by a 40 mL saline flush. Scanning was initiated when contrast opacification was observed in the descending thoracic aorta to capture a systemic arterial (bronchial artery-predominant) enhancement phase. Iodine maps were generated using Syngo VIA (Version VB60, Siemens Healthcare) with the virtual unenhanced (VUE) workflow, enabling separation of iodine and water components to facilitate identification of perfused (iodine-avid) regions versus relatively non-perfused regions consistent with necrosis. Table 1 summarises the DECT acquisition and iodine map parameters.

### 2.5. Iodine Map-Guided Target Selection Workflow

Immediately prior to biopsy planning, iodine maps were reviewed side-by-side with conventional CT images to identify the most perfused/viable intralesional component. Iodine density was quantified using region-of-interest (ROI) measurements placed within multiple visually distinct intralesional regions while avoiding adjacent enhancing structures, vessels, and artefacts. The biopsy target was selected in the region demonstrating the highest iodine density and regions with low iodine density were avoided when consistent with suspected necrosis. In our workflow, areas with an iodine density below 1.1 mg/mL were avoided. Previous studies have shown that iodine density values above 0.5 mg/mL indicate viable tissue and that this threshold distinguishes viable, enhancing tissue from non-enhancing or necrotic tissue on DECT [16,17,18].

Needle trajectory planning prioritised safe access to the iodine-avid target region while minimising traversal of fissures and emphysematous lung areas and avoiding major vessels and critical structures when feasible.

### 2.6. CT-Guided Biopsy Technique and Post-Procedure Assessment

Biopsies were performed under CT fluoroscopic guidance using a coaxial technique. CT-guided biopsies were performed by four specialists (two interventional radiologists and two pulmonologists) with approximately 30 years of experience for the most senior operator and 5–6 years of experience for the remaining operators in CT-guided thoracic biopsy. Local anaesthesia was administered with 2% lidocaine. Core needle sampling was performed using an Achieve™ automatic biopsy needle (CareFusion, San Diego, CA, USA), with needle lengths ranging from 11 to 20 cm and a gauge of 18–20 G, selected according to lesion depth and location. The usual number of passes was two per procedure. No on-site cytopathology (rapid on-site evaluation) was available.

### 2.7. Study Outcomes

The primary outcome of the study was diagnostic yield, defined as the proportion of CT-guided biopsies that yielded a definitive histopathological diagnosis. Secondary outcomes included the adequacy of biopsy samples for subsequent molecular testing, such as next-generation sequencing and Immuno-phenotyping, as well as the procedure’s safety profile, assessed by the incidence of complications, including pneumothorax and bleeding. Additionally, the study evaluated the feasibility of using DECT iodine maps to guide biopsies by targeting iodine-avid, viable regions within necrotic or heterogeneous lesions.

## 3. Results

Twenty patients underwent dual-energy CT-guided biopsy of necrotic thoracic lesions. The mean age was 56.6 ± 18.6 years (range 19–86 years), with 12 males and eight females. Median lesion long-axis diameter was 7.0 cm (range 2.0–11.0 cm) and mean thickness 5.4 ± 1.9 cm. The mean distance from the pleural surface to the lesion was 2.4 ± 1.7 cm (range 0–7.7 cm), and the total skin-to-lesion distance averaged 6.1 ± 2.2 cm. Most lesions (18/20) showed central necrosis on pre-biopsy imaging. Table 2 summarises patient and lesion characteristics.

Overall, 18/20 biopsies (90%) yielded a definitive diagnosis. Diagnostic yield varied by location: all mediastinal (8/8) and pleural (1/1) biopsies were diagnostic, whereas 9/11 pulmonary biopsies (81.8%) yielded diagnostic tissue. Diagnoses included non-small-cell lung cancer in eight cases (44.4%), classical nodular Hodgkin lymphoma in four cases (22.2%), thymoma in three cases (16.7%), and one case each (5.6%) of melanoma, breast carcinoma, and T-cell lymphoma.

**Figure 1:**
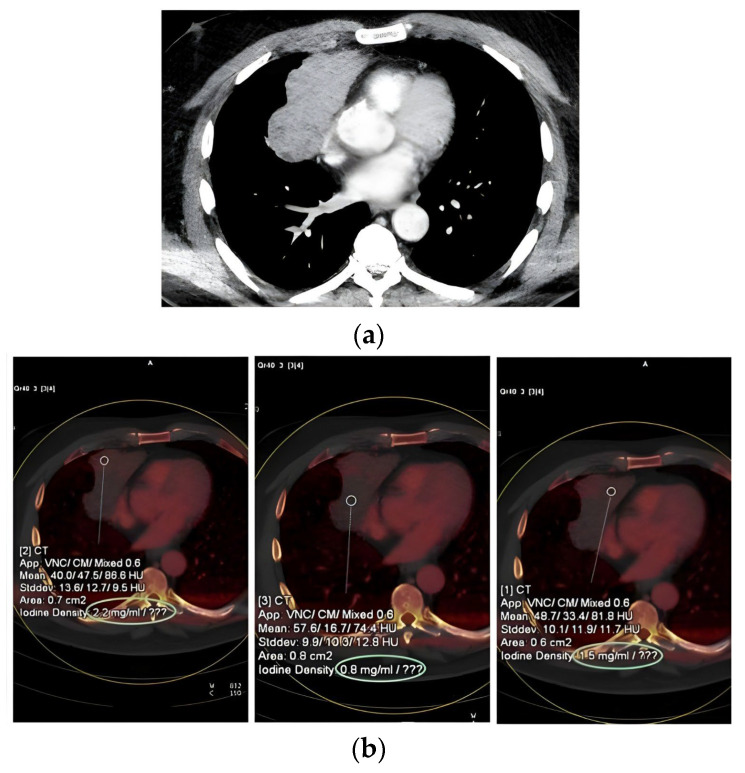
**Targeting viable regions in a necrotic anterior mediastinal mass using iodine mapping.** A 59-year-old male presenting with chest pain, cough, and B symptoms. (**a**) Contrast-enhanced axial CT image (**upper** panel) demonstrating a mildly heterogeneous anterior mediastinal mass. (**b**) Corresponding post-processed iodine maps (**lower** panels) demonstrating spatial heterogeneity in iodine density within the lesion; ROIs were used to quantify iodine concentration and guide targeting. Biopsy planning prioritised regions with iodine density ≥1.1 mg/mL (platform-specific threshold), favouring the highest iodine-avid areas while avoiding low-iodine/necrotic components. Histopathology confirmed a type B2 thymoma.

A representative case is presented to illustrate the practical application of DECT iodine mapping for biopsy planning in a heterogeneous lesion. Figure 1 demonstrates how iodine mapping was used to identify the most viable, iodine-avid region within a heterogeneous mediastinal mass for targeted biopsy.

**Figure 2:**
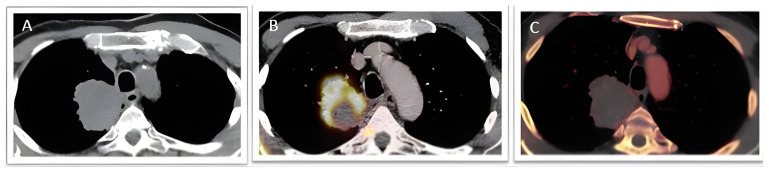
**Multi-modal imaging of a necrotic lung mass in a 60-year-old female.** A 60-year-old woman was referred for biopsy after the detection of a right upper lobe mass on a low-dose axial CT scan without contrast (**A**), performed during evaluation for chronic cough and unintentional weight loss. She underwent a pre-procedural PET scan (**B**), which showed heterogeneous peripheral uptake within the lesion. Due to the lesion’s large size and non-uniform tracer uptake, a dual-energy CT (DECT) iodine map (**C**) was obtained. The post-processed iodine map demonstrated patchy areas of variable contrast enhancement, indicated by a red colour in the heat map, representing viable, iodine-avid tissue, which were targeted for biopsy.

Figure 2 demonstrates a representative case in which DECT iodine mapping (C) identified viable tumour regions that were not evident on conventional low-radiation CT (A), thereby guiding a successful biopsy. The mass showed heterogeneous peripheral uptake on Fluorodeoxyglucose (FDG) PET/CT (B).

Adverse events were minor, with only one pneumothorax that did not require drainage and two mild bleedings, which stopped spontaneously.

Tissue adequacy for additional molecular testing such as Polymearse chain reaction (PCR) or next-generation sequencing (NGS) was achieved in 13 cases (65%). Additional testing was most common in mediastinal lesions (6/8, 75%) and least frequent in pulmonary lesions (6/11, 54.5%); the single pleural biopsy was also adequate for NGS. Among pulmonary lesions, NGS was performed in five cases and PCR in one; in mediastinal lesions, PCR was performed in five and NGS in one (Table 3).

Categorical variables are presented as frequencies and percentages. I 5 patients had their biopsy sent for NGS (next-generation sequencing), and three others had undergone a lobectomy with the NGS sent after surgery. II Immuno-phenotyping was performed on 3 cases of Hodgkin lymphoma. III Immuno-phenotyping was performed for T-cell lymphoma. IV BRAF staining was performed. V Staining for hormonal markers and Fluorescence in situ hybridisation (FISH) for HER2 mutation.

## 4. Discussion

The diagnosis and management of lung cancer, particularly NSCLC, remain complex and resource-intensive in clinical practice, often requiring timely histologic confirmation and adequate tissue for ancillary testing. CT-guided biopsy (CTGB) is a central tool in the diagnostic pathway and has consistently demonstrated high diagnostic performance. Across prior studies, reported diagnostic yields range from 71.2% to 97.6%, with sensitivity for malignancy between 85.7% and 97.3%, and specificity approaching 100% in selected cohorts [19,20,21].

However, CTGB performance is not uniform and may be reduced by lesion- and procedure-related factors, including smaller lesion size, challenging location, and intralesional necrosis/heterogeneity, which increase sampling error and the risk of non-diagnostic specimens. In particular, small lesions (≤2 cm) are associated with lower success rates [22]. Additionally, pneumothorax or intralesional heterogeneity, particularly necrosis, can further compromise the accuracy of tissue sampling [20,23].

Our study aimed to address one of these key limitations—targeting viable tumour tissue within necrotic lesions—by integrating DECT iodine mapping into real-time CT-guided biopsy. This approach enabled precise localisation of perfused, iodine-avid areas within masses that otherwise appeared heterogeneous or necrotic on standard CT. Notably, we achieved a 90% diagnostic yield, well within the high end of previously reported CTGB performance but in a uniquely challenging subset of patients with necrotic or complex lesions.

Compared to standard CTGB, which relies solely on morphological characteristics to guide needle placement, DECT adds a layer of functional imaging that can help clinicians avoid non-viable, necrotic zones. This was particularly relevant in our cohort, where standard CT alone may have failed to differentiate viable from non-viable tissue adequately. As such, our findings suggest that DECT-guided biopsy may help mitigate some of the key factors known to reduce diagnostic yield in conventional CTGB—especially when lesion architecture is heterogeneous.

Moreover, our results show that in over half of the patients, enough viable tissue was obtained for molecular and immunohistochemical profiling. This aligns with the needs of modern oncologic care, where molecular diagnostics and biomarker testing are integral to guiding therapy [24,25]. In contrast, necrotic tumour components frequently yield non-informative molecular results because necrosis is associated with degradation of nucleic acids and proteins, reduced DNA/RNA integrity, and lower tumour cellularity, which together can translate into assay failure, lower sequencing quality, and unreliable variant detection [26,27]. By facilitating targeting of iodine-avid, enhancing regions while avoiding non-enhancing/necrotic areas, DECT-guided sampling is therefore well positioned to mitigate one of the most common practical barriers to actionable molecular diagnostics in lung cancer.

Beyond the improved diagnostic yield, the safety profile of DECT-guided biopsies also merits attention. The low complication rate observed in our study, with only one pneumothorax and two minor bleedings, also supports the safety of integrating DECT into biopsy workflows. This is consistent with the safety profile of standard CTGB [28,29], further reinforcing DECT’s feasibility.

Prior research has demonstrated the role of DECT in improving tissue characterisation and targeting viable tumour areas in bone or soft-tissue metastases [13]. DECT is also increasingly used in oncology for lesion detection, therapy response evaluation, and vascular mapping [15]. However, to our knowledge, this is the first study to apply DECT iodine mapping in a clinical, real-time setting for CT-guided lung or mediastinal biopsy targeting necrotic lesions.

Despite the promising results of this proof-of-concept study, several limitations should be acknowledged. First, the study was conducted at a single tertiary care centre with a relatively small sample size (n = 20), which may limit the generalizability of the findings. In addition, iodine quantification and thresholds may be influenced by acquisition phase, vendor-specific DECT hardware and post-processing algorithms; therefore, our platform-specific workflow should be validated across additional systems before broader adoption. Second, there was no control group of patients undergoing standard CT-guided biopsy without DECT guidance, restricting our ability to draw direct statistical comparisons between DECT-guided and conventional CTGB; a randomised or matched cohort will be necessary to confirm any superiority. Third, comparative imaging—including virtual 120 kVp reconstructions, recent diagnostic contrast-enhanced CT, or FDG-PET—was not available for the vast majority of patients in this retrospective cohort, preventing systematic comparison of iodine maps with standard or metabolic imaging. Future prospective studies will incorporate standardised comparative imaging protocols.

Moreover, interpretation of iodine maps and target selection were performed by experienced clinicians in a high-volume centre, which may not reflect practices in other institutions, and inter-observer variability was not assessed. Additionally, although iodine uptake is a reasonable surrogate for perfusion and potential viability, it is not a definitive marker of cellularity or tumour burden; studies correlating DECT-derived parameters with quantitative histopathology are warranted. Finally, cost-effectiveness and workflow integration of DECT-guided biopsy were not evaluated and should be addressed in future research.

## 5. Clinical Implications and Future Directions

Necrosis and intralesional heterogeneity are frequent causes of sampling error in CT-guided biopsy, and conventional CT attenuation may not reliably differentiate viable tumours from non-viable components [20,23]. Our findings suggest that integrating DECT iodine maps into the targeting workflow is feasible and provides a physiologic dimension—regional perfusion/iodine uptake—that can complement morphologic assessment when selecting the biopsy trajectory. If confirmed in larger cohorts, this approach could translate into fewer non-diagnostic biopsies, improved tissue adequacy for molecular and immunophenotypic analyses, and fewer repeat procedures in lesions with suspected necrosis. Future work should include prospective controlled validation against standard CT guidance (ideally randomised or at least protocolized with blinded outcome assessment), with pre-specified criteria for “necrotic/heterogeneous” lesions and standardised reporting of the number of passes, core length/quality, and complication definitions. It would also be valuable to assess the incremental role of virtual monoenergetic imaging (VMI)—either alone or combined with iodine maps—in improving conspicuity and tissue contrast for target selection. Finally, multi-centre, multi-vendor validation is needed to confirm the reproducibility of iodine quantification and targeting decisions across different DECT platforms and reconstruction pipelines.

In conclusion, DECT-guided CT biopsy may represent a meaningful evolution of the standard CTGB technique, particularly in patients with necrotic or complex thoracic lesions. By improving target selection and avoiding non-viable tissue, DECT may enhance diagnostic yield, facilitate molecular testing, and ultimately improve the quality of care. Larger, multi-centre trials will be essential to validate these results and assess cost-effectiveness. Still, our findings provide an important proof of concept for integrating functional imaging into interventional biopsy planning.

## Figures and Tables

**Table 1:** DECT acquisition and iodine map reconstruction protocol.

Parameter	Protocol
Scanner	SOMATOM^®^ Dual Source Force™ (Siemens Healthineers, Forchheim, Germany)
Acquisition mode	Dual-source DECT
Tube potential	90 kVp (fixed)
Tube current	24/46 to 390/60 mAs (dual-source range)
Rotation time	250 ms
Pitch	0.55
Contrast agent	Iohexol 80–85 mL + saline flush 40 mL
Timing	Scan initiated at contrast opacification in descending thoracic aorta (systemic arterial/bronchial phase targeting)
Post-processing software	Syngo VIA VB60 (Siemens Healthcare)
Iodine map method	VUE workflow (iodine–water separation)
Radiation metrics recorded	CTDIvol and DLP (mean DLP reported in text)

**Table 2:** Patient demographics, biopsy sites, and procedure-related complications.

	Result
Procedures, n	20
Male gender, n (%)	12 (60)
Female gender, n (%)	8 (40)
Age (mean + SD)	56.6 ±18.6
Lesion size, cm (mean + SD)	7.0 ± 2.9
Lesion thickness, cm (mean + SD)	5.4 ± 1.9
Distance from the pleural surface to the lesion, cm (mean + SD)	2.4 ± 1.7
Total skin-to-lesion distance, cm (mean + SD)	6.1 ± 2.2
Biopsy location:	
Anterior mediastinum, n (%)	8 (40)
Right upper lobe, n (%)	6 (30)
Right middle lobe, n (%)	1 (5)
Right lower lobe, n (%)	2 (10)
Left upper lobe, n (%)	2 (10)
Pleura, n (%)	1 (5)
Complications:	
Pneumothorax, n (%)	1 (5)
Bleeding, n (%)	2 (10)

Categorical variables are presented as frequencies and percentages. Normally distributed continuous variables are presented as mean ± SD; SD, standard deviation.

**Table 3:** Diagnostic yield and availability of additional molecular testing.

Diagnosis	Number of Cases	Cases with Sufficient Material for Additional Testing
Non-small-cell lung cancer, n (%)	8 (40)	5 (67.5) ^I^
Hodgkin lymphoma, n (%)	4 (20)	3 (75) ^II^
Thymoma, n (%)	3 (15)	2 (66.67)
T-cell lymphoma, n (%)	1 (5)	1 (100) ^III^
Melanoma, n (%)	1 (5)	1 (100) ^IV^
Breast carcinoma, n (%)	1 (5)	1 (100) ^V^

Categorical variables are presented as frequencies and percentages. ^I^ 5 patients had their biopsy sent for NGS (next-generation sequencing), and three others had undergone a lobectomy with the NGS sent after surgery. ^II^ Immuno-phenotyping was performed on 3 cases of Hodgkin lymphoma. ^III^ Immuno-phenotyping was performed for T-cell lymphoma. ^IV^ BRAF staining was performed. ^V^ Staining for hormonal markers and Fluorescence in situ hybridisation (FISH) for HER2 mutation.

## Data Availability

The datasets used and/or analysed during the current study may be obtained from the corresponding author upon reasonable request, with the requisite permission from the Institutional Review Board of Rabin Medical Center—Beilinson Hospital.

## Abbreviations

List of Abbreviations: BRAF—B-Raf proto-oncogene; CT—Computed tomography; CTGB—CT-guided biopsy; DECT—Dual-energy CT; FDG—Fluorodeoxyglucose; FISH—Fluorescence in situ hybridization; G—Gauge; HER2—Human epidermal growth factor receptor 2; IHC—Immunohistochemistry; NGS—Next-generation sequencing; NSCLC—Non-small-cell lung cancer; PET—Positron emission tomography; ROI—region of interest; SD—Standard deviation; SCLC—Small-cell lung cancer; VUE—Virtual unenhanced.
